# Chondroma of the urinary bladder: A case report

**DOI:** 10.1097/MD.0000000000029229

**Published:** 2022-05-27

**Authors:** Kuo-Wei Kao, Weiming Cheng, Yi-Chin Chang

**Affiliations:** a Division of Urology, Department of Surgery, Taipei City Hospital, Renai Branch, Taipei, Taiwan; b Division of Urology, Department of Surgery, Taipei City Hospital, Zhongxiao Branch, Taipei, Taiwan; c Institute of Biopharmaceutical Science, School of Life Science, National Yang Ming Chiao Tung University, Taipei, Taiwan; d Department of Urology, Faculty of Medicine, National Yang Ming Chiao Tung University, Taipei, Taiwan; e Program in Molecular Medicine, School of Life Sciences, National Yang Ming Chiao Tung University, Taipei, Taiwan; f Division of Pathology, Taipei City Hospital, Zhongxiao Branch, Taipei, Taiwan.

**Keywords:** benign neoplasms, cartilaginous, case report, chondroma, urinary bladder neoplasms

## Abstract

**Rationale:**

Chondromas are benign tumors comprising cartilaginous tissue that commonly occur in the small bones of the hands and feet. Chondromas are extremely rare in extraskeletal soft tissues, and only six cases of bladder chondromas have been reported thus far.

**Patient concerns:**

A 75-year-old woman presented with abdominal pain and urinary symptoms, including increased frequency and a weak stream.

**Diagnosis:**

Cystoscopy revealed a well-defined bladder mass over the anterior bladder wall. The pathology report showed neoplastic chondrocytes within the hyalinized and focal myxoid matrix, and immunopositivity for S-100, leading to the seventh known diagnosis of bladder chondroma.

**Interventions:**

The tumor was endoscopically resected. The postoperative stay was uneventful, and 5 days later, the patient was discharged after the removal of the urinary catheter.

**Outcomes:**

One month after surgery, repeated cystoscopy showed no recurrence of the bladder tumor, and the patient reported improvement in urinary symptoms and relief of lower abdominal pain.

**Lessons:**

Chondromas of the urinary bladder can present as urinary symptoms and abdominal pain in older patients. Transurethral resection is the treatment of choice.

## 1 Introduction

Chondromas are benign cartilaginous tumors that mostly affect skeletal tissues, especially short bones of the hands and feet.^[1]^ Extraskeletal soft tissue chondromas are extremely rare, and only 6 cases of bladder chondroma have been reported previously.^[2–7]^ Bladder chondromas may cause urinary symptoms, abdominal pain, or hematuria. We report the case of a 75-year-old woman in whom bladder chondroma was discovered incidentally and managed by transurethral resection. To the best of our knowledge, this is the first case of bladder chondroma diagnosed in Asia.

## 2 Case presentation

**Figure 1:**
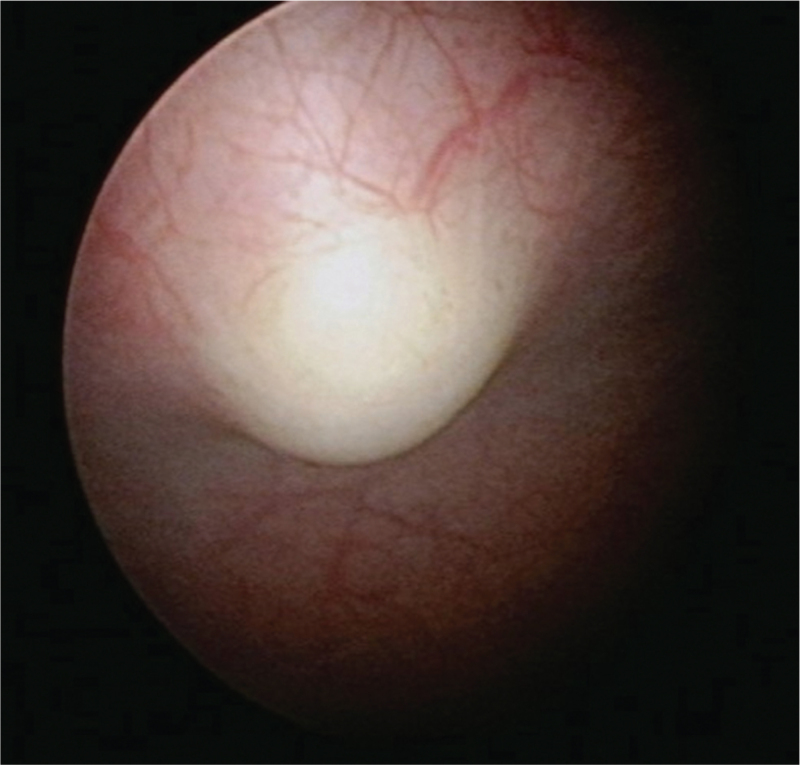
Bladder chondroma showing a well-defined and smooth appearance under cystoscopy.

A 75-year-old woman presented with the chief complaints of lower abdominal pain, increased urinary frequency, and a weak urine stream. She had a medical history of diabetes mellitus and hypothyroidism, both of which were controlled with prescribed medications. She reported increased urinary frequency, weak stream, lower abdominal pain on bladder distention, and dysuria for 2 months. Urinary analysis revealed no hematuria or pyuria. A video urodynamic study was performed to rule out detrusor underactivity and bladder outlet obstruction, which showed normal compliance of the urinary bladder and no bladder outlet obstruction. A tentative diagnosis of interstitial cystitis was made, and cystoscopy and hydrodistension were performed under general anesthesia. A 2-cm wide, well-defined, submucosal broad-based mass was found at the anterior bladder wall (Fig. 1). The bladder mass was completely endoscopically resected. The postoperative stay was uneventful, and 5 days later, the patient was discharged after the removal of the urinary catheter.

**Figure 2:**
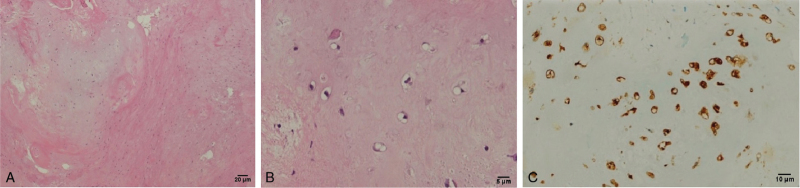
(A) The tumor growing in a lobular pattern, comprising neoplastic chondrocytes within the hyalinized and focal myxoid matrix (×40). (B) Neoplastic chondrocytes with small, dark nuclei within the lacunae (×200). (C) Presence of neoplastic chondrocytes confirmed by the immunopositivity for S-100 (×100).

Microscopic examination of the resected tumor mass revealed a lobular pattern of growth with neoplastic chondrocytes scattered within the hyalinized and focal myxoid matrix. Neoplastic chondrocytes comprised small dark nuclei within the lacunae, and the lesion was confirmed by immunopositivity for S-100 (Fig. 2A–C). Additionally, the tumor base was not involved in the tumor tissue.

One month later, repeated cystoscopy showed no recurrence of the bladder tumor, and the patient reported improvement in urinary symptoms and relief of lower abdominal pain.

## 3 Discussion

Chondromas are rare benign tumors comprising mature cartilage that mostly occur in bones.^[8]^ Skeletal chondromas are often asymptomatic but may sometimes cause swelling and pain depending on the location of the lesion.^[9]^ Soft tissue chondromas are located in the extraosseous and extrasynovial areas. Most of these tumors are painless and grow slowly. Approximately 64% of soft tissue chondromas have been reported to occur in the hands.^[10]^ The genetic variations associated with soft tissue chondromas remain largely unknown. A previous study revealed translocation involving chromosomes 2 and 13 in extraskeletal chondrosarcoma.^[11]^ Another study reported that soft tissue chondroma was related to monosomy 6 and rearrangement of chromosome 11.^[12]^ Furthermore, translocation of chromosomes 8, 12, and 13 and clonal numerical changes have been reported.^[13,14]^ The recurrence of soft tissue chondroma is low after local excision, and no malignant transformation has been reported.^[15]^

Chondromas of the urinary bladder are extremely rare. Differential diagnoses include papilloma, urothelial carcinoma, squamous cell carcinoma, leiomyoma, paraganglioma, fibroma, plasmacytoma, rhabdomyosarcoma, and leiomyosarcoma.^[16]^ In the existing literature, only 6 cases of bladder chondromas have been reported. A noteworthy fact is that all the reported patients, including the one in our case, are women, and the age at diagnosis is between the sixth and eighth decade. The presenting symptoms of bladder chondromas include abdominal pain and urinary symptoms such as frequent urination, dysuria, and hematuria. Cystoscopic examination revealed a whitish submucosal mass with a smooth, well-defined surface. Interestingly, all bladder chondromas reported to date were located at the anterior wall or bladder dome^[2–7]^ (Table 1). Transurethral resection is an effective management strategy for bladder chondromas, and no recurrence has been reported following this procedure. To the best of our knowledge, this is the first diagnosed case of bladder chondroma in Asia. This case suggests that bladder chondroma should be suspected as a possible diagnosis in older women presenting with abdominal pain and increased urinary frequency, and cystoscopy must be performed if medical treatment fails. Although chondroma of the urinary bladder is rare, it can occasionally present with urinary symptoms and abdominal pain in older women. Transurethral resection is the treatment of choice.

**Table 1:** Published cases of bladder chondroma.

Case no.	Author, year	Age (years)	Sex	Country	Symptoms sign	Tumor location	Pathological features	Management	Disease status
1	Pauwels et al,^[2]^	63	Female	Belgium	Abdominal pain	Ventral dome	Embedded in a chondroid matrix.S100, vimentin positive	Transurethral resection	Disease-free
2	Perrino et al,^[3]^	62	Female	USA	Dysuria, frequency, nocturia, abdominal pain	Anterior wall	Mature hyaline cartilage within the lamina propria. S100, vimentin positive	Transurethral resection	Disease-free
3	Carter et al,^[4]^	54	Female	Canada	Right lower quadrant pain	Anterior wall	Lobulated, bland, hypocellular, mature hyaline cartilage and fragments of muscularis propria. S100, vimentin positive	Transurethral resection	Disease-free
4	Tamayo-Jover et al,^[6]^	67	Female	USA	Microscopic hematuria	Anterior wall	Benign urothelium with nodular fragments of bland mature hyaline cartilage. S100, vimentin positive	Transurethral resection	Disease-free
5	Tazeh et al,^[5]^	72	Female	Spain	Microscopic hematuria	Anterior wall	Nodular and lobulated tumor composed of hyaline cartilage, without cellular atypia. S100, vimentin positive	Transurethral resection	Disease-free
6	Ngweso et al,^[7]^	65	Female	Australia	Urgency, nocturia	Right anterior wall	Mature cartilage with chondrocytes in the lacunae and abundant chondromyxoid stroma	Transurethral resection	Disease-free
7	The presented case, 2021	75	Female	Taiwan	Abdominal pain, frequency, weak stream	Anterior wall	Neoplastic chondrocytes scattered within the hyalinized and focal myxoid matrix, S100 positive	Transurethral resection	Disease-free

## Author contributions

**Conceptualization:** Kuo-Wei Kao.

**Data curation:** Kuo-Wei Kao.

**Software:** Weiming Cheng.

**Validation:** Yi-Chin Chang.

**Visualization:** Yi-Chin Chang.

**Writing – original draft:** Kuo-Wei Kao.

**Writing – review & editing:** Kuo-Wei Kao, Weiming Cheng.
